# Correlation between night sweats and season fluctuation in China

**DOI:** 10.3389/fpubh.2024.1423698

**Published:** 2025-01-09

**Authors:** Cai Chen, Meng Li, Fulai Peng, Danyang Lv, Xiao Ding, Chongxuan Tian, Weilian Ren, Xiangwei Meng, Tiefeng Sun, Yi Wang, Haitao Du, Fengxia Wu, Wei Li, Ping Wang

**Affiliations:** ^1^Shandong Institute of Advanced Technology, Chinese Academy of Sciences, Jinan, China; ^2^The Second Affiliated Hospital of Shandong University of Chinese Medicine, Jinan, China; ^3^Affiliated Hospital of Shandong University of Traditional Chinese Medicine, Jinan, China; ^4^Biomedical Engineering Institute, School of Control Science and Engineering, Shandong University, Jinan, China; ^5^Jinan Children's Hospital (Children's Hospital Affiliated to Shandong University), Jinan, China; ^6^Shandong Academy of Chinese Medicine, Jinan, China; ^7^School of Basic Medical Sciences, Shandong University, Jinan, China

**Keywords:** night sweat, season, Internet search index, Baidu index, time series decomposition, wavelet transform

## Abstract

**Background:**

Night sweats are a condition in which an individual sweats excessively during sleep without awareness, and stops when they wake up. Prolonged episodes of night sweats might result in the depletion of trace elements and nutrients, affecting the growth and development of children.

**Purpose:**

To investigate the relationship between sweat nights and season.

**Method:**

The Internet search index for night sweats in Zhengzhou, China was obtained from the Baidu index during 2011–2022. Meteorological factors, including ambient temperature, humidity, pressure, precipitation and average wind speed in Zhengzhou were obtained from the website https://en.tutiempo.net/climate. A time series decomposition model was used to study the relationship between night sweats and seasonality. Continuous wavelet transform and cross wavelet transform were utilized to explore the relationship between night sweats and meteorological factors.

**Result:**

A typical periodic pattern is evident in the seasonal trend. Specifically, following a peak in January each year, there is a rapid decline followed by a secondary peak, after which a trough occurs. The search index of night sweats increased rapidly in the first stage, slowed down in the second stage, and showed negative growth from 2011 to 2014. The correlation coefficients between the search index for night sweats and atmospheric pressure as well as average temperature, are 0.25 and-0.26, respectively.

**Conclusion:**

Night sweat was connected with season. Specifically, the number of night sweat search index increased in the cold season and declined in the summer season. Night sweat was negatively connected with temperature but favorably correlated with air pressure.

## 1 Introduction

Night sweats occur when someone sweats excessively while sleeping and stops when they wake up (1). The defining feature of this condition is a significant rise in sweating during overnight sleep, which stops once the individual awakens, as if perspiration had sneakily emerged while the person was oblivious. Persistent sweating at night could lead to significant loss of water and electrolytes, such as sodium, potassium, and magnesium, which are vital for maintaining overall health and normal physiological functions. Prolonged episodes of night sweats might result in the depletion of trace elements and nutrients, affecting the growth and development of children and reducing the physical fitness and immunity levels in adults. Night sweat often disrupt sleep, causing individuals to wake up frequently, which affects the continuity and depth of their sleep (2). Over time, this could end up in issues such as fatigue, lethargy, difficulty concentrating, and memory loss. James W Mold et al. found that night sweats were linked with daytime fatigue (OR = 1.99, 95% CI: 1.12–3.53), and waking pain at night (OR = 1.87, 95% CI:1.16–2.99) (2). Retrospective review based on two sleep laboratories in Oklahoma City showed that compared with those who did not report night sweats, patients with night sweats were more likely to suffer from daytime fatigue (*p* = 0.001), snoring (*p* = 0.003), and breathing trouble (*p* < 0.0001) (3). Night sweats could be an indication of underlying health conditions such as tuberculosis (4), hyperthyroidism (5), coronary heart disease (6) and climacterium (7).

A systematic review of the literature revealed that in older adult primary care patients, the prevalence of night sweats was 10%, while in obstetric inpatient wards, the prevalence among women was 60% (8). As for children, the study by So HK et al. found that among 6,381 children (with a median age of 9.2 years, ranging from 7.7 to 10.7 years), 3,225 were boys (50.5%) (9). Within the past 12 months, 747 children (11.7%) experienced night sweats weekly, and boys were more likely to have night sweats than girls.

A cross-sectional study found that 10% of 795 primary care patients reported having experienced night sweats and perceived a correlation between night sweats and factors such as age, muscle spasms, and numbness in their hands and feet (10). A longitudinal study involving 11,725 women found that 7% of the population reported experiencing frequent night sweats, and this subgroup had over double the likelihood of developing coronary heart disease, with an odds ratio (OR) of 2.38 and a 95% confidence interval (CI) ranging from 1.62 to 3.50 (6).

With the rapid advancement of internet technology and mobile internet, as well as a significant increase in public health awareness, a growing proportion of people seek relevant information online when they experience illness or suspected symptoms, with the goal of understanding the causes of their conditions, finding treatment methods, accessing drug information, or seeking medical advice. For example, when a symptom such as “night sweats” shows frequently in people’s search queries, the Baidu Index produces related search trends. The Baidu Index largely reflects the level of attention internet users devote to keyword search patterns on the Baidu search engine, allowing us to better understand how certain phrases, such as “night sweats” change over time, including seasonal swings. In theory, by studying differences in the search volume for the keyword “night sweats” throughout different seasons on the Baidu Index, one might indirectly determine whether public interest in night sweats rises or falls with the seasons. Right now, there is no concrete evidence linking night sweat to season. In this study, we made use of Baidu Index data to investigate the relationship between night sweats and seasonal changes.

## 2 Method

**Figure 1:**
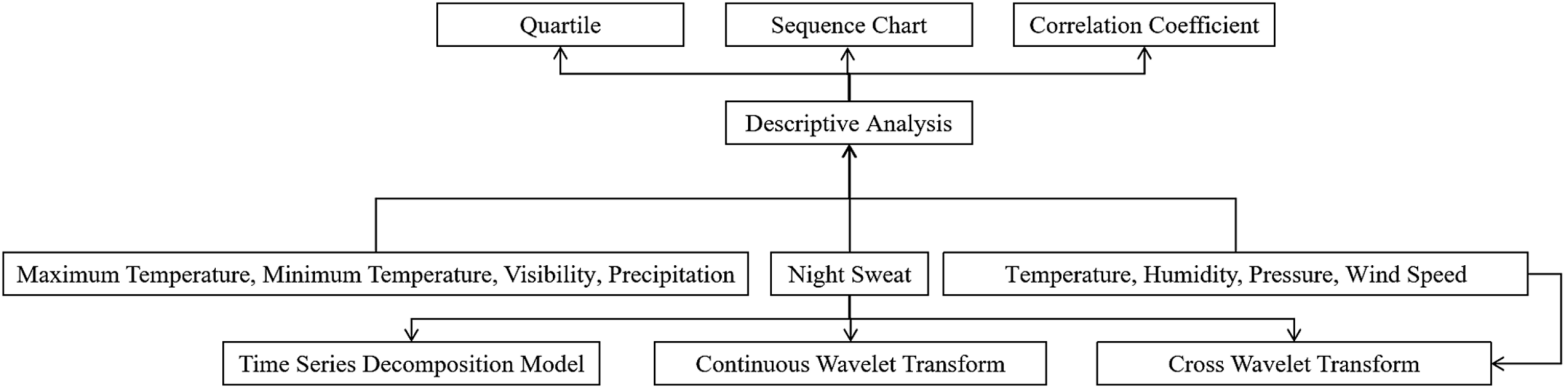
A flow chart of methodology.

Figure 1 depicts the experimental approach used in this investigation. First, a descriptive analysis is performed on the search index for night sweats and climatic parameters, which includes correlation coefficients and time series line charts. Second, a time series decomposition model is used to investigate the seasonal changes in night sweats. The autocorrelation of night sweats is analyzed using a continuous wavelet transform. The cross-wavelet transform is used to investigate the relationship between night sweats and temperature, humidity, air pressure, and wind speed.

### 2.1 Data

Internet search index for night sweats was from Baidu index.^1^ Baidu Index is a data analysis platform established by the Baidu search engine that utilizes huge volumes of user behavior data. It enables users to analyze keyword search trends and acquire insights into internet users’ interests and demands. However, due to the popularity of the Baidu search engine in China, research related to the Baidu Index is only available in China. In our early research, we used the Baidu Index to generate a search map for asthma in China and investigate the association between snoring and seasons (11, 12). This illustrates that it is possible to conduct disease dispersion studies using search engine data.

Keyword ‘night sweats’ was typed into the main interface of Baidu Index, and then the research region was setting to all the country, and research period was setting from 2011 January to December 2022. Also, in order to investigate the relationship between night sweat and meteorological factor, including ambient temperature, maximum temperature, minimum temperature, visibility, humidity, pressure, precipitation and average wind speed, Zhengzhou capital of Henan province, China, was selected as research area. Internet search index about night sweat in Zhengzhou was collected from Baidu Index from 2011 to 2022. Contemporaneous meteorological factors in Zhengzhou were obtained from the website https://en.tutiempo.net/climate.

### 2.2 Time series decomposition model

A time series decomposition model was used to study the relationship between night sweat and seasonality, as shown in Equation 1 (13).


(1) $$Y_{t}=T_{t}+S_{t}+E_{t}$$

Here, $Y_{t}$ represents the time series data, which in our study is the Internet search index for night sweat from Baidu index. $T_{t}$ , $S_{t}$ , and $E_{t}$ denoted the time trend, seasonal trend, and random variable, respectively. Time series decomposition models was consist of two inner loops and one outer loop. The inner loops were primarily used for decomposing the trend component and the seasonal component of the time series data. The inner loop was calculated as follows (14, 15).

1. Remove the trend

The trend component $T_{t}^{\mathrm{trend}}$ from the previous iteration $T_{t}^{k}$ was removed from the time series data (Equation 2).


(2) $$T_{t}^{\mathrm{trend}}=Y_{t}-T_{t}^{k}$$

Where *k* represents the k-th iteration.

1. Seasonal smoothing

LOESS smoother was used to smooth the periodic series $T_{t}^{\mathrm{trend}}$ to obtain the initial seasonal component $S_{t}^{k+1}$ .

1. Low-pass filtering

A low-pass filtering was used to smooth the periodic series $S_{t}^{k+1}$ to compute $S\prime_{t}^{k+1}$ and LOESS smoother were utilized to smooth the $S\prime_{t}^{k+1}$ to obtain $T\prime_{t}^{k+1}$ .

1. Seasonal component

Seasonal component $S_{t}^{k+1}$ was obtained according to Equation 3.


(3) $$S_{t}^{k+1}=S\prime_{t}^{k+1}-T_{t}^{\prime k+1}$$

1. Deseason. A seasonally adjusted component $S_{t}^{de-\mathrm{season}}$ was calculated according to Equation 4.

(4) $$S_{t}^{\left(de-\mathrm{season}\right)}=Y_{t}-S_{t}^{\left(k+1\right)}$$

1. Trend smoothing.

A LOESS smoother was used to smooth the $S_{t}^{de-\mathrm{season}}$ obtained in Step (5) to get trend trend component $T_{t}^{k+1}$ .

During the outer loop process, the random variable is calculated as follows according to Equation 5.


(5) $$E_{t}^{k+1}=Y_{t}-S_{t}^{k+1}-T_{t}^{k+1}$$

### 2.3 Wavelet transform

Wavelet transforms are extremely useful in the study of medical time series data, particularly when dealing with non-stationary, complicated dynamic properties, and weak signals. They have been widely employed in various medical time series analyses, such as electrocardiogram analysis, sleep staging, and epilepsy prediction. In this work, nocturnal sweat data is fundamentally a time series signal; thus, wavelet analysis is used to predict disease cycles and analyze condition-related variables.

#### 2.3.1 Continuous wavelet transform

The definition of wavelet basis function is shown in Equation 6. $\psi_{a,b}\left(t\right)$ is the continuous wavelet basis function obtained by scaling and translation, where a is the scale factor and b is the scale translation factor (16, 17).


(6) $$\psi_{a,b}\left(t\right)=\frac{1}{\sqrt{a}}\psi \left(\frac{t-b}{a}\right),a>0,b\in R$$

Continuous wavelet transform is defined as Equation 7. ${WT}_{x}\left(a,b\right)$ represents


(7) $${WT}_{x}\left(a,b\right)=\frac{1}{\sqrt{a}}\int \times \left(t\right)\bar{\psi }\left(\frac{t-b}{a}\right)dt$$

${WT}_{x}\left(a,b\right)$ is the continuous wavelet transform result of $x\left(t\right)$ , and $x\left(t\right)$ is the Internet search index data of night sweats in this study. As shown in Equation 7, the one-dimensional time series signal can be transformed into a two-dimensional signal in time-frequency domain by wavelet transformation. The signal’s time-frequency analysis could be performed by modifying the stretching factor (a) and transfer factor (b).

#### 2.3.2 Cross wavelet transform

The cross wavelet spectrum between two time series x(t) and y(t) can be defined as Equation 8 (18, 19), where $C_{x}\left(\alpha ,\tau \right)$ is the wavelet transform coefficient of the sequence x(t), and $C_{y}^{*}\left(\alpha ,\tau \right)$ is the complex conjugate of the wavelet transform coefficient of the sequence y(t). The cross wavelet spectrum can reflect the region where the periodic intensity of two sequences is consistent, allowing users to determine the degree of correlation between the two sequences in several simultaneous frequency domains.


(8) $$W_{xy}\left(\alpha ,\tau \right)=C_{x}\left(\alpha ,\tau \right)*C_{y}^{*}\left(\alpha ,\tau \right)$$

## 3 Result

### 3.1 Descriptive results

**Figure 2:**
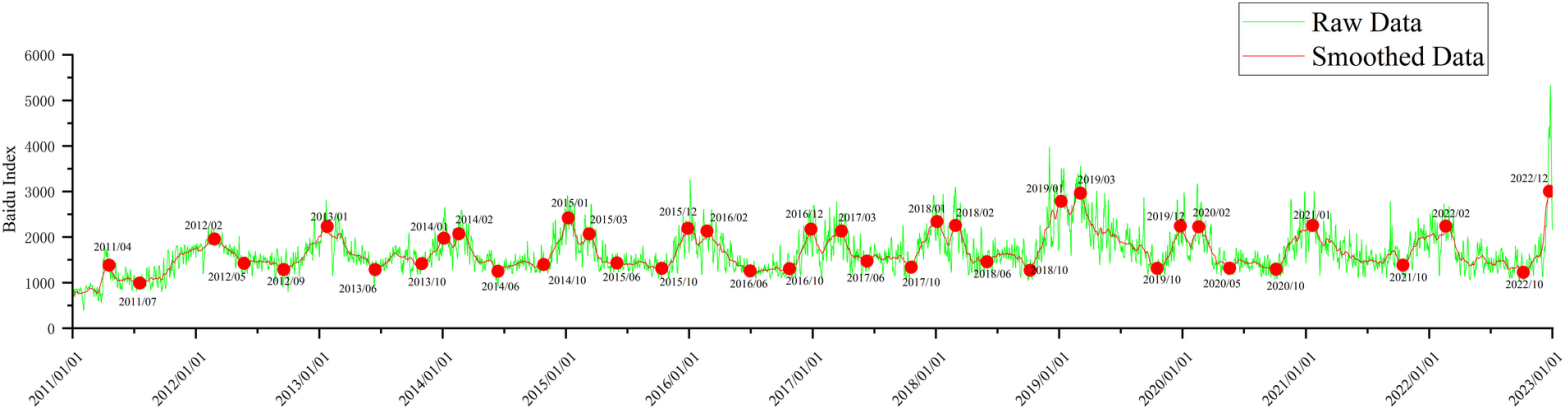
Internet search data for night sweats from January 2011 to December 2022 (green line, raw data; red line, smooth data).

Figure 2 illustrates Internet search data for night sweats from January 2011 to December 2022 sourced from Baidu Index. The green line depicts the raw data, while the red line represents the smoothed data. The graph shows that the search index about night sweats grew in September or October and peaked in February or March of the following year. The search index thereafter began to fall, troughing from June to October with some regularity. The raw data (green) increased dramatically in December 2022 (during the COVID-19 pandemic in China). From 2014 to 2020, there will be twin peaks every year. The peak for 2019–2021 is smaller than that of 2020.

**Figure 3:**
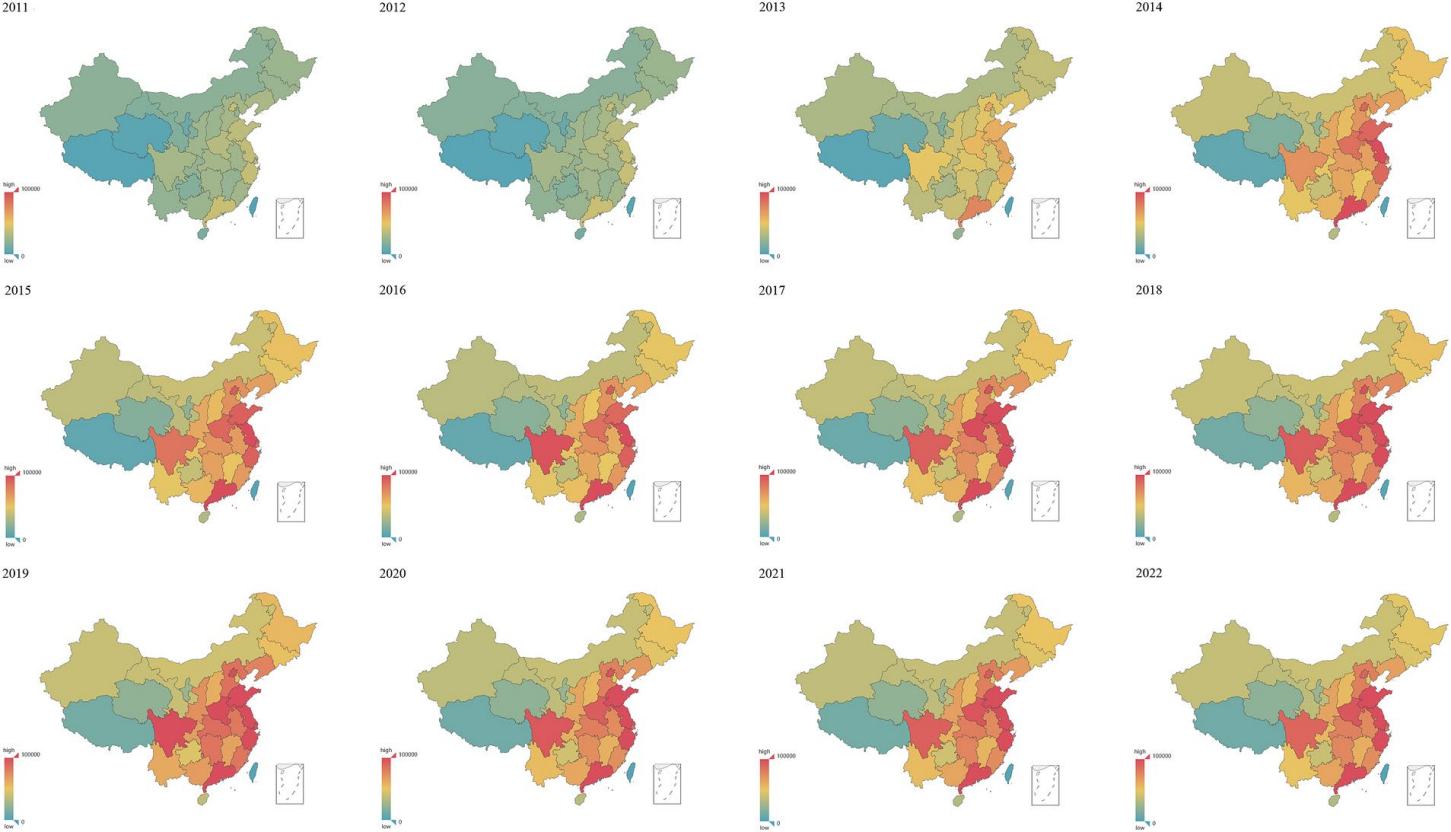
Geographical distribution of the search index for night sweats in China between 2011 and 2022.

Figure 3 demonstrates the geographical distribution of the search index for night sweats in various provinces between 2011 and 2022. In 2011, there was little attention paid to night sweats across the country; from 2011 to 2015, interest progressively increased; however, in 2015 and beyond, people’s attention to night sweats peaked. Night sweats are often more of a worry in economically developed communities along the eastern coast than in the inland. Sichuan Province (in southwest China’s heartland) is more worried about night sweats than other inland locations. Southern Chinese provinces are more worried about night sweats than northern ones.

**Figure 4:**
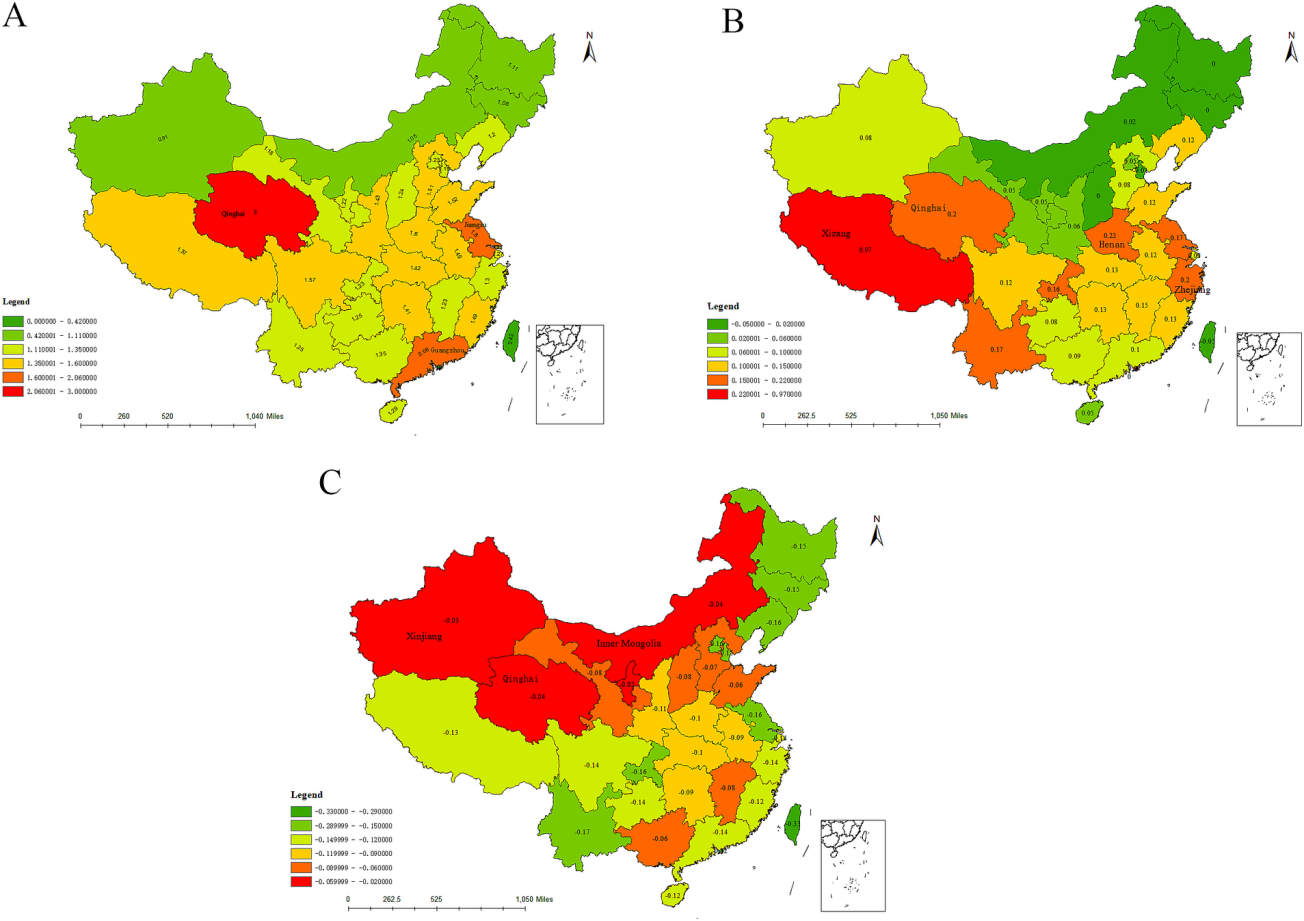
Growth of internet search index for night sweats (**A** from 2011 to 2014; **B**, from 2015 to 2018; **C**, from 2019 to 2022).

Figure 4 describes the growth of the Internet search index for night sweats in three phases: 2011 to 2014, 2014 to 2018, and 2019 to 2022. In general, the search index of night sweats increased rapidly in the first stage, slowed down in the second stage, and showed negative growth in the third stage. As shown in Figure 4A, during the first stage(from 2011 to 2014), the Internet search index for night sweats in Qinghai, Guangzhou, and Jiangsu provinces demonstrated the most rapid growth, with respective growth rates of 3, 2.06, and 1.8, respectively. During the second stage (from 2015 to 2018), the Internet search index growth for night sweats in Tibet, Henan, Qinghai, and Zhejiang provinces was the fastest, respectively, showing increases of 0.97, 0.22, 0.2, and 0.2, respectively. As for the third stage, the growth rate of all provinces about night sweats in the country was negative.

**Figure 5:**
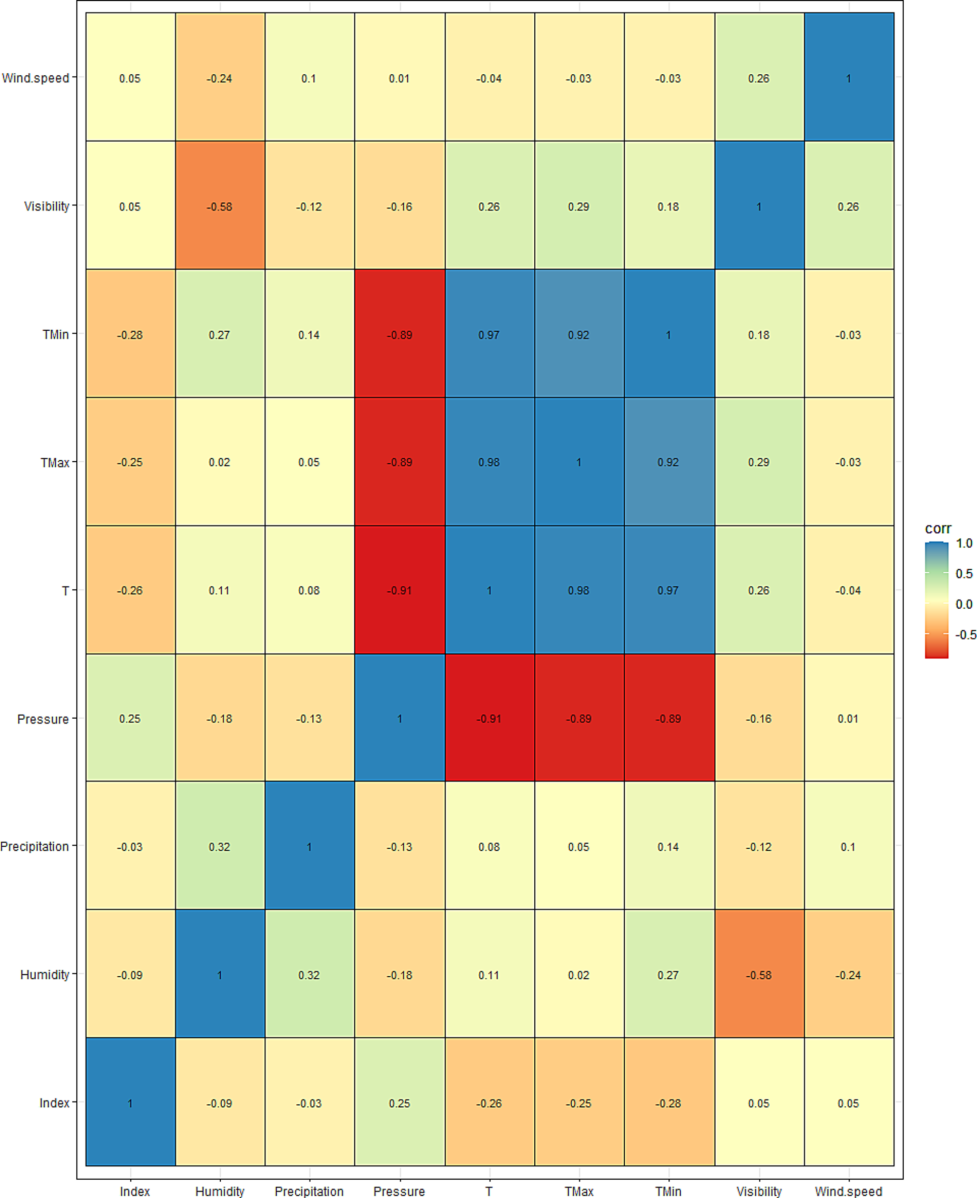
Correlation coefficients between the night sweat search index and meteorological factors (index, Internet search index for night sweat; T, daily average temperature; Tmax, maximum daily temperature; Tmin, minimum daily temperature).

**Figure 6:**
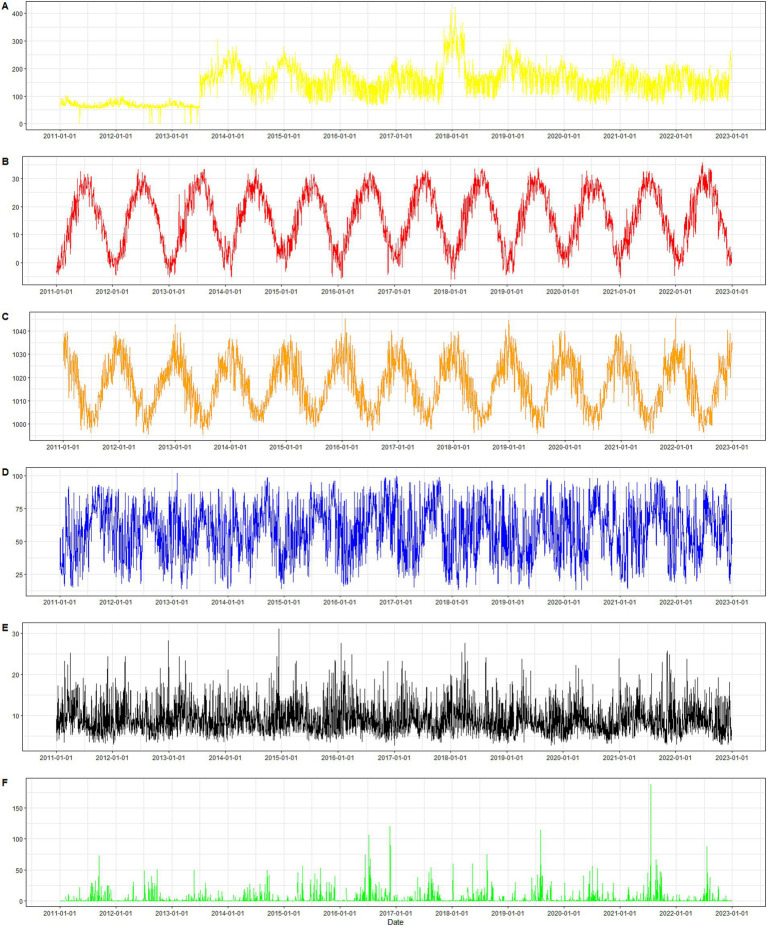
Time series plot from 2011 to 2022. **(A)** The night sweat search index; **(B)** ambient temperature; **(C)** humidity; **(D)** atmospheric pressure; **(E)** average wind speed; **(F)** precipitation.

Table 1 shows the descriptive results of the Internet search index about night sweats and ambient temperature, humidity, atmospheric pressure, average wind speed and precipitation in Zhengzhou from 2011 to 2022. Maximum ambient temperature, precipitation and atmospheric pressure were 35.7°C, 188.72 mm, and 1045.6 hPa. Figure 5 depicts the correlation coefficients between the night sweat search index and meteorological factors. The correlation coefficients between the night sweat search index and humidity, precipitation, air pressure, average temperature, maximum temperature, minimum temperature, visibility, and average wind speed are distributed as follows: −0.09, −0.03, 0.25, −0.26, −0.25, −0.28, 0.05, and 0.05, respectively. Figure 6 illustrates a time series plot from 2011 to 2022 for the night sweat search index (Figure 6A), ambient temperature (Figure 6B), humidity (Figure 6C), atmospheric pressure (Figure 6D), average wind speed (Figure 6E) and precipitation (Figure 6F) in the Zhengzhou. Temperature, humidity and night sweat search index all showed periodic changes, and the trend of temperature and night sweat search index is opposite.

**Table 1:** Descriptive results of the internet search index about night sweats and meteorological factors from 2011 to 2022.

	Temperature	Pressure	Humidity	Precipitation	Wind speed	Index
Min	−5.9	995.5	13	0	2.8	0
Q1	7.2	1007.725	43	0	6.5	92
Q2	16.7	1017.1	59	0	8.3	153
Q3	24.9	1025.175	73	0	10.7	185
Max	35.7	1045.6	102	188.72	31.1	422

Index, Baidu index for night sweats.

### 3.2 Result of time series decomposition

**Figure 7:**
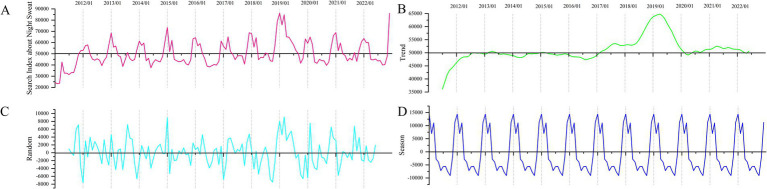
Results of the time series decomposition model. **(A)** Internet search index for night sweat; **(B)** long-term trend; **(C)** random variables; **(D)** random variables.

The results of the time series decomposition model are shown in Figure 7. Figure 7A depicts the overall time series change of night sweats from 2011 to 2022. Time series decomposition models decompose the series into long-term trend (Figure 7B), random variables (Figure 7C), and seasonal trends (Figure 7D). From 2017 to 2019, the basic trend exhibited an upward trajectory before experiencing a sharp drop to around 5,000 in 2019. The index generally fluctuated around a baseline value of approximately 5,000 during other periods. In 2019, a sudden elevation occurred in the random variable, causing interference in the waveform of the time series data (Figure 7C). As shown in Figure 7D, a typical periodic pattern is evident in the seasonal trend. Specifically, following a peak in January each year, there is a rapid decline followed by a secondary peak, after which a trough occurs.

### 3.3 Wavelet transform

**Figure 8:**
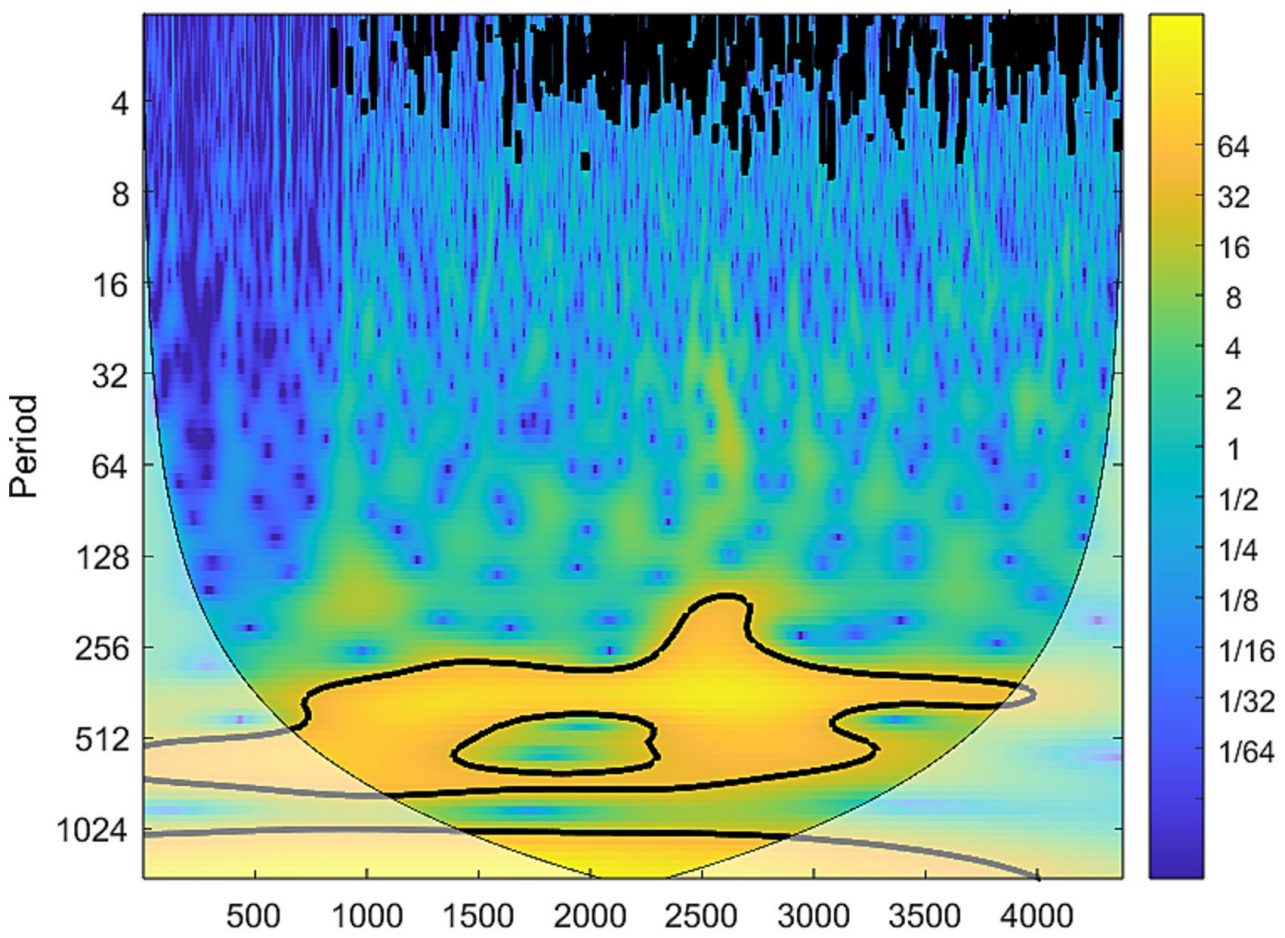
Continuous wavelet transform for the night sweat search index (black line, 95% confidence interval).

**Figure 9:**
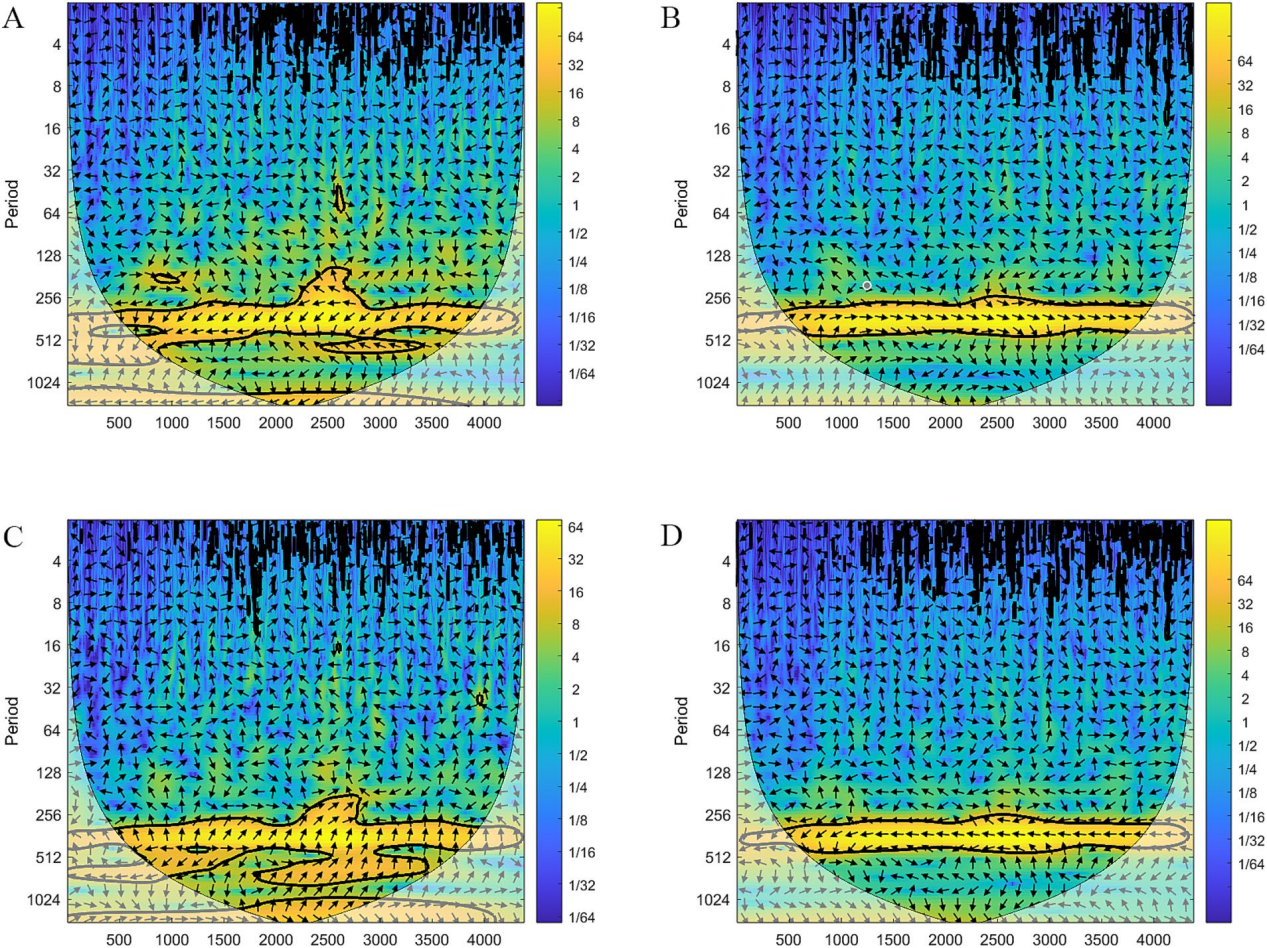
Cross-wavelet spectrum between the night sweat index and meteorological factors. **(A)** Humidity; **(B)** pressure; **(C)** wind speed; **(D)** ambient temperature; black line, 95% confidence interval; arrow to the right, positive correlation; arrow to the left, negative correlation.

The results of the continuous wavelet transform for the night sweat search index are presented in Figure 8. The horizontal axis represents days, with January 1, 2011 designated as day 1 and December 31, 2022 as day 4,383. The vertical axis denotes the period, and colors closer to yellow indicate higher energy levels. From the figure, a concentration of yellow energy is observed around a period of approximately 365 on the vertical axis, suggesting the presence of a 365-day oscillatory pattern. Figure 9 illustrates the cross-wavelet spectrum of the night sweat index against humidity, air pressure, wind speed, and temperature. The black lines represent the influence cones, within which lies the region of effective spectral values. The thick black outline indicates the boundary of the 95% confidence interval. The arrow to the right indicates the same direction change with a positive correlation. The arrow to the left indicates the opposite change, the negative correlation. As shown in Figure 9B, the arrows pointed to the right, which means that air pressure was positively correlated with the search index for night sweats. On the opposite, the arrows pointed to the left in Figure 9D, which means that ambient temperature was negatively associated with the search index for night sweats.

## 4 Discussion

Based on the night sweats search index and the time series decomposition methodology, we observed that night sweats were seasonal. The number of search index for night sweats grew in the cold season but decreased in the summer. Furthermore, cross-wavelet analyses revealed that night sweat was adversely associated to temperature but positively related to air pressure.

We suspect the following factors may be involved. Sweating control is complicated, involving both thermoregulatory and nonthermoregulatory systems. When the core body temperature rises above a certain limit or threshold, perspiration helps to bring it down (8). When a core body temperature threshold is reached, a hypothalamus response is triggered, which activates thermoregulatory mechanisms such as sweating (20, 21). This might happen as a result of decreased external heat exposure or heat dissipation (for example, too much clothes or bed cover) or increased heat generation (for example, excessive muscle activity). In the winter, individuals typically utilize heating equipment such as heaters and electric blankets to stay warm, resulting in much higher inside temperatures than outdoor temperatures. Excessive indoor temperatures may make it difficult for the body to release heat while sleeping, and the thermoregulatory mechanism activates the sweating response to reduce body temperature, resulting in night sweats. Furthermore, wearing heavy clothing and warm bedding in the winter can easily raise the skin’s surface temperature above the comfort threshold, and the human body excretes surplus heat through sweating, contributing to nocturnal sweats.

Our findings contradict another finding. A study of the relationship between temperature, season, lifestyle and experiences of hot flashes and night sweats (HFNS) in middle-aged women in the United Arab Emirates found that temperature and seasonal temperature variations did not appear to influence HFNS reports (22). We hypothesize that the reason could be due to the varied effects of the two locations’ climates. The United Arab Emirates, located near the equator, has a tropical desert climate that is dry and hot for most of the year. This differs substantially from China, which has a temperate monsoon climate with four distinct seasons and a wide temperature range. Furthermore, we discovered that air pressure is positively connected with night sweats, indicating that the search index for night sweats is particularly large when air pressure is high, which is consistent with earlier research. International menopausal research of climate, altitude, temperature, and vasomotor symptoms found that hot flashes and night sweats were more common at low altitudes (where air pressure is relatively high) than at high altitudes (23). It’s possible that air pressure is linked to diseases like tuberculosis (24). Tuberculosis is one of the diseases for which night sweats are a primary symptom (25).

Most women have night sweats, which are one of the vasomotor symptoms of menopause. According to studies, 60–80% of women suffer night sweats at some point throughout the menopausal transition, with the incidence and frequency increasing in late perimenopause and early postmenopause, or within a few years before and after their last menstrual period (26). Night sweats during menopause may be directly linked to hormonal changes. Randolph JF Jr. et al. discovered that the prevalence of vasomotor symptoms (night sweats or hot flashes) increased with (log) follicle-stimulating hormone (FSH) concentration, and FSH concentration is positively connected with the frequency of hot flashes or night sweats (27). Gold EB et al. found that vasomotor symptoms significantly decrease with increasing estrogen concentrations (28). According to published studies, seasons might influence hormone levels in the body (29). Xu SR et al. discovered that in cold seasons, the expression of follicle-stimulating hormone (FSH) associated to estrogen reduces in domesticated yaks (30). This shows that during the cold season, women’s estrogen levels may drop, increasing vasomotor symptoms like night sweats. Nicolau GY et al. discovered that thyroid-stimulating hormone (TSH) levels are lower during cold seasons, but plasma thyroid hormone concentrations peak at the same time. Lower TSH levels and higher plasma thyroid hormone concentrations may lead to increased perspiration (31). In addition to gender variations, night sweats may be connected with age. In a study of 822 patients with obstructive sleep apnea, Arnardottir ES et al. discovered that night sweats were strongly associated with younger age (32). Under similar settings, age and age-related traits might influence the pace at which body heat is gained and the methods by which it is lost. When Inbar O et al. investigated thermoregulatory responses, they discovered that, while the final average skin temperature changes were similar across three age groups (children, adults, and the older adult), children had the lowest heat storage compared to adults and the older adult (33). In contrast, prepubescent children had the largest net metabolic heat output compared to body weight and heat intake from the environment. The fundamental physical difference between children and adults in terms of thermoregulation is that children have a significantly larger surface area-to-mass ratio, resulting in higher rates of heat absorption or loss (34).

The highlight of our study is that it provides evidence based on internet search behavior of the correlation between night sweats and seasons, and proposes an analysis of the degree of correlation using cross-wavelet transform. We must acknowledge the limitations of this study. As the Baidu search engine is widely popular in China, the use of the Baidu Index is limited to China. Because the data is derived from online search patterns, differences in users’ degrees of digital literacy may induce bias. Individuals’ understanding and capacity to use technology varies, which might affect their effectiveness in looking for, analyzing, and utilizing online material, consequently influencing the gathering and interpretation of study data. Furthermore, variations in internet availability among regions are a significant limiting factor. Not all geographic locations have equal access to high-quality internet connections; certain isolated or impoverished places may lack reliable internet connectivity. This disparity in availability can exclude particular individuals, resulting in sample selection bias and perhaps failing to fully represent the realities of locations with limited internet access. Finally, user search behavior is a complex process influenced by personal preferences, prior knowledge, and information requirements. Different search habits and tactics may yield diverse results, affecting the consistency and general application of the research findings. For example, some people may prefer certain search engines or websites while ignoring other potential sources of information. In conclusion, these factors may have an impact on the research outcomes.

## 5 Conclusion

Night sweats were connected with seasons. Specifically, night sweats search indexes increased in the cold season and declined in the summer. Night sweats were negatively associated with temperature but favorably correlated with air pressure.

## Data Availability

The original contributions presented in the study are included in the article/supplementary material, further inquiries can be directed to the corresponding authors.

## Footnotes

^1^ http://index.baidu.com
